# An Estimation of the Effect of Green Financial Policies and Constraints on Agriculture Investment: Evidences of Sustainable Development Achievement in Northwest China

**DOI:** 10.3389/fpubh.2022.903431

**Published:** 2022-07-12

**Authors:** Bingjing Mei, Arshad Ahmad Khan, Sufyan Ullah Khan, Muhammad Abu Sufyan Ali, Jianchao Luo

**Affiliations:** ^1^College of Economics and Management, Northwest A&F University, Xianyang, China; ^2^Shaanxi Rural Financial Research Center Yangling, Xianyang, China; ^3^Department of Economics and Finance, UiS Business School, University of Stavanger, Stavanger, Norway; ^4^International Business School, Shaanxi Normal University, Xi'an, China

**Keywords:** green finance, difference-in-differences approach, agriculture investment, heterogeneity of farmers, sustainable development, Northwest China

## Abstract

Farming' community actively participating as micro-actors in green finance schemes is critical for regional planning and development. On the basis of the extent to which financial progress and sustainable development are coordinated, in a difference-in-differences approach, this article employed 2350 small investigations to estimate the influence of green-finance strategies on peasants' agriculture investment and developed a mediation effect method. It investigates the role of peasant managerial variability in mediating the influence of financial constraints. The results indicate that the introduction of a financial restriction variable reduces the positive impacts of green-finance regulations on peasants' agricultural investment. Moreover, peasants who participate in non-agricultural management exercises are more inclined to take advantage of green financing regulations and are affected via financial restrictions in mediate means. The building of a green-finance sector in remote regions should accomplish unique positioning and rapid growth.

## Introduction

Supporting integrated rural economic and environmental sustainability is a problem for regional development of China (1). It is indeed critical for China, like a significant international resources consumption and CO_2_ emission (2, 3), to achieve the green and low-carbon agricultural revolution. This necessitates the shift of agricultural operations from a conventional resource-intensive mode to a green and cost-effective mode. It is necessary to invest in technical advancement, operational mechanisms, and the accurate application of suitable financial resources to green farming activities (4).

Finance seeks to benefit the actual economy by allocating assets rationally. Green finance, generally relates to economic operations that promote green betterment, deal with climatic changes, and realize conservation of resources and effective exploitation, arises as a necessity (5). China recently issued top-level design principles for green finance development and studied the construction of a green-financial scheme. Its progression has been remarkable, since its recommendation in 2015 to its implementation in 2016. A multi level green-financial market system with a inclusive green-financial policy arrangement has presented. Green-financing policies (GFPs) have been implemented in agriculture and village areas. GFPs serve a key role in changing agro industrial base, rising the proficiency levels of agrarian green products, and promoting financial progress (6, 7). Rural regions, on the other hand, not focus only on GFP's promotional regions, as well as its weakness. Despite investing in green finance initiatives is expanding in China, it is on a micro level and is scattered (8). This cannot fulfill the demand of green agriculture's diverse expansion (9). Furthermore, due to the low profits and considerable risks associated with green agricultural programs, green financial services are given less attention. These obstacles have deterred farming producers from seeking green financing assistance. It enhances the financial restrictions and reduces the provision of GFPs to assist farmers' green agriculture productivity. The creation of a green financial sector in rural regions should accomplish distinctive orientation and leapfrogging progression, and it must be backed by quality science.

Producers' voluntary participation is the foundation for GFPs' practical deployment in agricultural regions. Unlike several researchers (10, 11), who use organizational data to investigate GFPs' effect on green technology innovation and energy use efficiency, fewer researchers (12–16) have examined at its impacts on crop investing from the perspective of farmers.

Simultaneously, finance has emerged as the most massive issue for growers throughout green-growth (17). Producers' production expenditure and technical advancement had hindered via financing constraints (18). Policy implementers employ “Selective Execution” to balance “Profit Target” and “Support Agriculture Task,” that increases financing constraint. Nevertheless, it has been small investigation on the effects of GFPs on farmers' financial limitations, and nothing on the mechanics of intermediate financial restrictions on GFP and agriculture investment in rural areas. Conferring to some scholars, peasant diversity would have an effect over green agricultural productivity and investment (19, 20). Producers' managerial scale and managerial status, for example, have a substantial influence on their acceptance of the green farming production method (21, 22). Producers' off-farm financial integration does have an economic impact on peoples living conditions and farming techniques acceptance (23, 24). The off-farm occupation increases their ability to gain new technology 2004), which impacts overall productive expenditures. Financing might reduce producers' productivity and expenditure pressures, as well as help with green agronomic funding. Farmers' credit availability is affected by farmers' off-farm employment behavior (25), and financing constraints will adjust according to the heterogeneity of farmers' employment types. There is a paucity of research in the context of improving GFPs' effectiveness, regarding the farmers of different employment types' responses to GFPs. There is limited research examining the impact of the heterogeneousness of farmers' management kinds over financing limitation mechanism.

The coordinated expansion of environment and finance (CEF) is a scholarly fact (26) that is required for the successful implementation of green finance methods. GFPs seeks to undertake the encounter among development and environmental protection, subsequent in high quality green economic growth (18). If an area's environmental and financial growth are well, it is desirable to build a green-finance strategy. It promotes the growth of green-finance. In this context, this research examines the influence of GFPs on producers' agriculture investment and develops a mediation effect model. Additionally, research investigates the impact of various farmer managerial systems on the intermediary effect of financial limitations.

It offers sufficient information for the application and improvement of GFPs in rural areas: (1) This research evaluates the effect of green finance policy on farmers' agricultural investment using farmers' innovative scientific study viewpoints. (2) Its significant addition is an examination of the relationship between GFPs and peasants' crop produce investment from the standpoint of financial limitations. (3) Focusing on the variability of farmers' managerial techniques, it examines the mediating effect of financial restrictions on GFPs and agricultural investment. It examines the real recipients of GFPs in rural regions, as well as the integration of farmers' environmental expenditures within particular capital constraints. It analyses if the additional agricultural production expenses resulting from the program will push out alternative production investment. It investigates if non-agricultural management may help farmers improve agricultural investment and encourage green production.

## Theoretical Framework and Hypotheses

GFP might encourage the reduction of obsolete production capability, as well as help in changing industry base and rising the efficiency levels of green-products (18). The GFP credit management framework includes environmental considerations. It might minimize the allocation of resource to energy-intensive and environmentally damaging activities (27) while encouraging green-technology research and investments via businesses (28). GFPs comprise first specific policy of China on green-finance, which is an essential step to economically promote the expansion of green agriculture (29). In theory, it might enhance peasants' productivity sources (30). It has the potential to enhance the efficiency of improved farming production and expansion of diverse financing approaches. Policy penetration in rural regions might have had an effect on peasants' agriculture output actions and finance techniques. Growers' investing in sustainable and low carbon production materials might well be accelerated, as well as producing technological updates encouraged. As a result, farmers' ecologically friendly production expenses rise.

The attitude of financial firms that effectively assist the funding of conservation of energy and protection of the environment initiatives demonstrates the application of GFPs. Firms use economics to tackle ecological threats by altering its financing system. GFPs lacking financial advantages as a result of the non-executive concept, and financial firms are hesitant to adopt green financial operations (31). Agriculture green initiatives typically involve extended life spans and significant capital investment. Several financial companies claim this green business is unprofitable and will limit institutions' financial outlook (32). As financial firms react to GFPs in order to increase capital stability and acquire diversified competitiveness (33), they are adhering to “green standard.” Financing firms are more likely to grant money to substantial protection of the environment, as well as green and energy-saving businesses with strong industrial operational standards (34). Usually, few sustainable investment goods available for medium and small environmental protection businesses, as well as households. This has a negative influence on GFP development in remote regions, as well as enhancing peasants' access to green financial institutions. Peasant's' financial restrictions are so exacerbated.

Farmers' actively participating as local operators in policy is critical. Numerous people in rural areas, though, face credit limits, that limit investment and accumulation of capital. Peasants who face financing constraints have lower tolerance for risk (35). People are vulnerable to the double restrictions of unpredictability and availability, that potentially hinder the optimum degree of allocating resources. This has an impact on agriculture green and low-carbon production factor. Peasants' reliance on less-costly borrowing has grown as agriculture production expenses have risen as a result of environmental restrictions. Peasant's' favorable regulatory actions could be hampered by consequent financial limitations, which can impede peasants' sustainable green investments. As a result, GFPs can only provide little recommendations on green agricultural growth.

Non-agricultural operations is now a significant income source for peasant (36) and has a promotional impact on the investment of peasant in green farming (23). Peasants' increasing agricultural investments is not only the result of environmental policy execution regulation and benefits; it seems to be a spontaneous process of adaptation in important financial factors. It would be influenced by variations in labor and material resources induced by peasants' non-agricultural operations. According to studies, non-farm incomes does have a beneficial impact on peasants' loan provision (23, 37). It could serve as an alternative for the credit requirement generated by investment in agriculture. Since non-agricultural capital formation boosts productivity of agriculture, the budget line of peasants' agricultural investment choices would be moved right side. Peasants' agriculture productivity investment decisions may be influenced by this. As a result, given budgetary restriction, peasants having non-agricultural management seem to be more inclined into becoming GFP grantees, with a profound effect with their individual agriculture productivity investment decisions.

**Figure 1:**
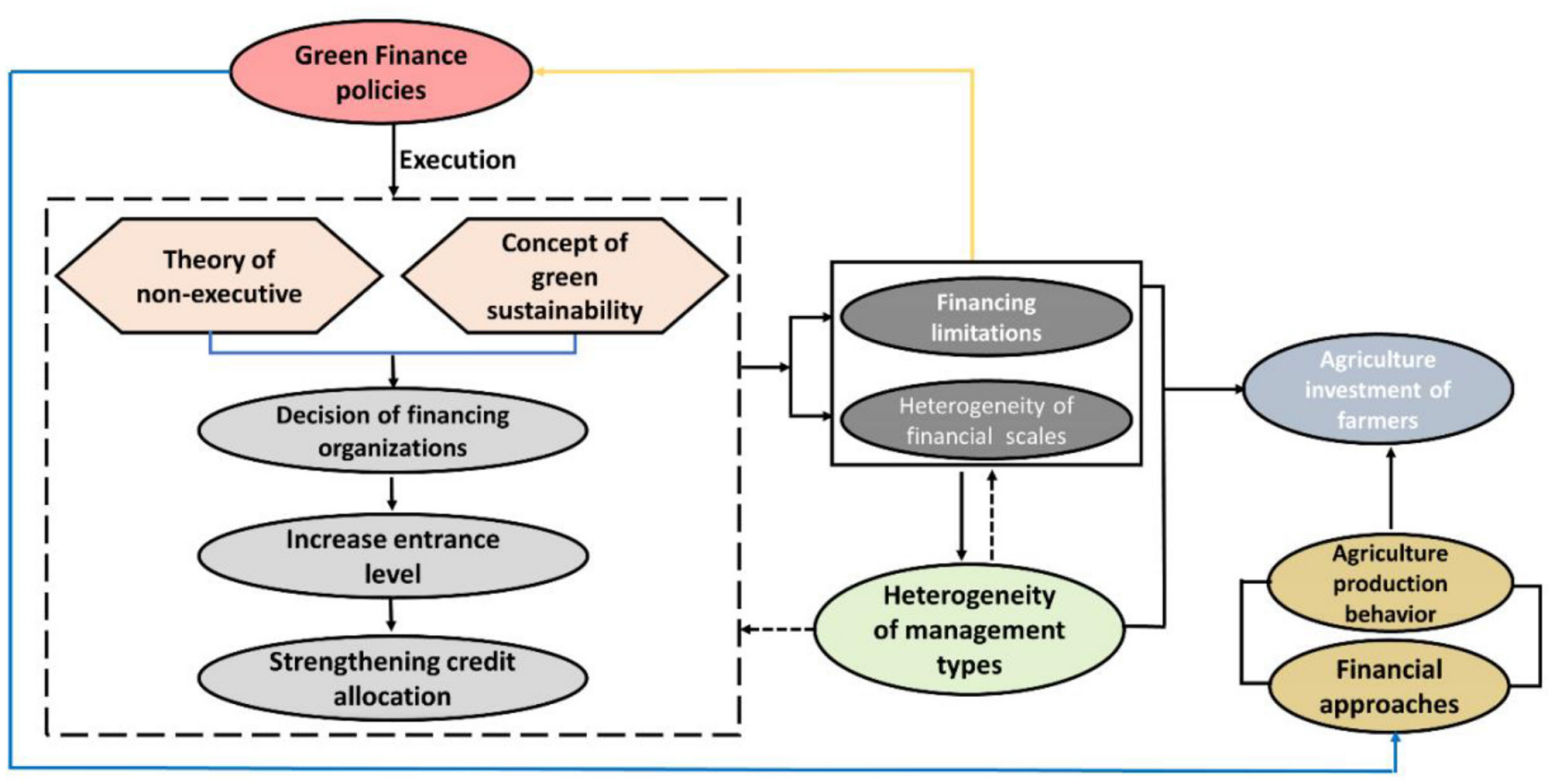
Agriculture investment of the farmers (mechanism of GFPs).

Figure 1 depicts an overview of the GFP's impact process on peasants' investment on agriculture. One of the prerequisites for the successful functioning of GFP is the integrated growth of the ecosystem and financing. The samples in the current research are classified based on their degree of financing development and environment sustainability. The preceding assumptions are suggested depending on the assumption that a region has a significant level of synchronized environmental and financial development;

H1: GFPs have a positive impact on agriculture investment by peasants.
H2: The nation encourages the notion of green development, and financial firms increase the Admittance Threshold of green financial services, resulting in financial restrictions for peasants.
H3: At the moment, the implementation of GFPs has had no significant effect on mitigating peasants' financial restrictions, and policies' directing role in encouraging peasants' agriculture investment is constrained.
H4: Peasants who engage in non-agricultural management practices are highly inclined to be GFP recipients and are impacted via financing restrictions in GFPs' intermediating framework over agriculture investment.

## Research Methodology

### Research Design

Regarding the lateness of GFPs and their low impact in rural regions, the current research considered the CFE as critical to their deployment. The micro-survey study samples are classified appropriately. In order to examine the impacts of GFPs over agriculture investment of farmers and to corroborate the sample groups rationality, the present research utilizes difference-in-differences (DID) approach. This research utilized the model of mediation effect to evaluate the probable mechanism of finance limitations between agriculture investment of farmers and GFPs, with farmers' finance scale heterogeneity which acts as an intermediary. Thus, based on this concept, farmers management heterogeneity's influence over financing restrictions' mediating impact is investigated in this research.

### Sampling Selection

The areas of western China lack inherent benefits in terms of infrastructure, capital and human resources when compared to rest of the areas. Nonetheless, efficient establishment of green finance in the areas of western China is a critical linkage in achieving the green economic revolution. In this regard, the academics and government both must pay close consideration to this issue.

The sampling sites were two provinces of western China, i.e., Ningxia Province and Shaanxi Province, which exhibit typical agriculture development characteristics. The methods of agriculture production of Shaanxi are simple and controlled by conventional farming due to the diverse geography and landscapes, as well as the little per capita agricultural land region. Southern and northern Shaanxi's geological environments are unstable, and soil degradation is a problem. The rate of urbanization in the region of Guanzhong is rising, and gap between supply and demand for aquatic resources is widening. Shaanxi, on the other hand, is one of the fastest-growing provinces in western China, with a strong basis for agriculture technical transitions and financing assistance. Ningxia Province, which is situated on northwest inland plain, has agriculture resources to its benefit. Whereas, it's hampered by aquatic resource scarcity and a vulnerable ecosystem. Ningxia has abundant and diversified grasslands resources, making animal husbandry growth favorable. Ningxia, in addition, has a variety of production techniques and agriculture sector benefits, although its agri-sector is characterized by massive management and refining is minimal.

**Figure 2:**
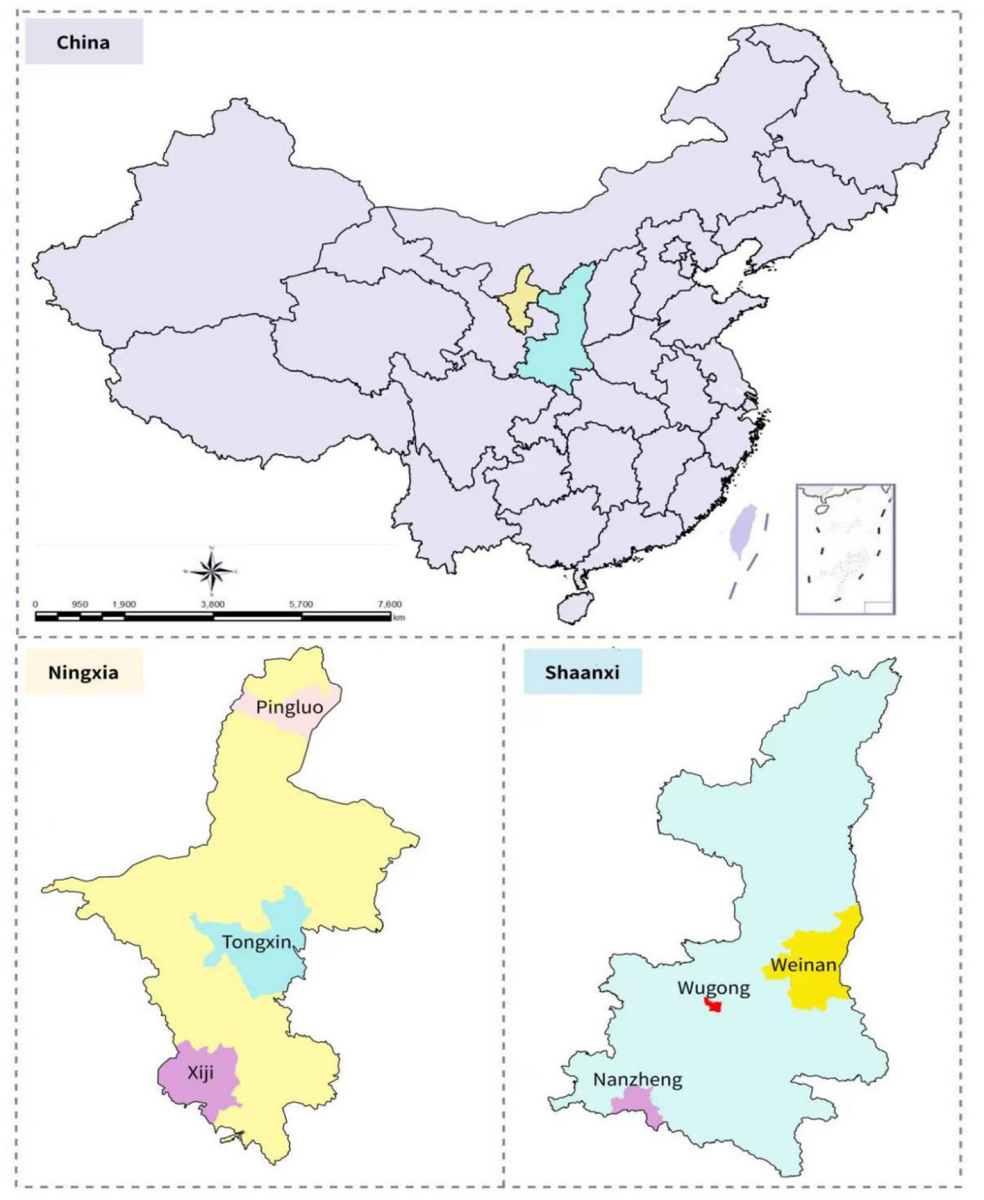
Study area map.

Their agriculture advancement confronts severe concerns as an important grain-producing regions of China. The agricultural labor- and land productivity both are incompatible. Both require to expand their existing and conventional agriculture revitalization and transition, as well as enhance their financing support. They must establish green capital/finance, execute the conversion, and shift to green agriculture from conventional agricultural system. The government of China in 2012 set out to foster all-round societal advancement in five sectors: social, cultural, political, economic, and environmental development. The green development premise has infiltrated every facet of social and economic growth. It issued guidance perspectives on the development of green finance system in 2016, as well as a list of key actions. The time node in the current research will be estimated 1 year after the deployment of policy begins to the target's impact, as per the recommended time of policy. Thus, GFP came into effect in 2017. Assuming the availability of data and lack of interfering incidents during the period of the study, setting the research period for 2013–2019 will yield the basic policies impact. The research was conducted using sample and questionnaire surveys. From 2013–2019, we collected panel data on the investment for agriculture production by farmers and loan payments from prescribed financing sectors in 6 districts of both Ningxia and Shaanxi Provinces (Figure 2). The missing data was removed, leaving the balanced panel data with 2,350 micro-observations.

### Empirical Model Specification

The current research used DID model to split the empirical and control groups by assessing the CEFs' degree in the sample. Farmers having high level of coordination and cooperation were in experimental group, whereas control group was the comparative. By taking into account geographical disparities in financial improvement and environmental sustainability, numerous interfering elements influenced the environmental measurement, therefore the index might be inaccurate. The current investigation utilized the model developed by Qiaoxin (38) (Equation 1) regarding coupling coordination degree, and applied the degree of financial-environment sustainability coupling as a proxy for the degree of environmental-financial coordination.


(1) $$\begin{array}{r} CEF=2\sqrt{\left(u_{1}\times u_{2}\right)/{\left(u_{1}+u_{2}\right)}^{2}}\times \left(0.5u_{1}+0.5u_{2}\right) \end{array}$$

The following are the methods in detail; (a) Using standard processes, identify the relevant finance-development and environment-sustainability assessment indices and their associated pattern values *u*_1_ and *u*_2_. (b) Identify the extent/degree of local environmental and financial coordination via the model of coupling coordination degree. The level of CEF is proportional to the degree of coupling coordination.

The ratio of local loan to deposit was used as a proxy variable to represent financial development from a scale point of view. Regional regulation of the environment was used as a proxy to measure environmental sustainability (39). The regulation of environment is an institutional tool that allows the authorities to exert direct control over the use of natural resources. The environmental management of the government was primarily concerned with the emission of pollutants (40), that directly related to the pollution management costs. Thus, the cost of emissions reduction will be lower for per unit industrial additional value if the regulatory authorities' efficiency is high and the environmental management of the regional government will be more stringent. The influence of industry structure was adjusted by taking into account the considerable changes in industrial mix between areas. By following (40, 41), the below Equation 2 is used for measuring the regulatory requirements for environment;


(2) $$\begin{array}{r} E_{it}=a_{it}P_{it}/Q_{it} \end{array}$$

In Equation 2, region and year are represented by *i* and *t*, respectively. Amount of investment and project for controlling the industrial pollution in the region are represented by *P*_it_. Industry additional value in the region is represented by *Q*_it_. Whereas, the share of industrial additional value (regional) in industrial additional value (national) is represented by a_it_. China Financial and Statistical Yearbooks, Ningxia and Shaanxi Statistical Yearbooks, and Shaanxi Regional Statistical Yearbook were used to compile the data for this research. Table 1 displays the degree of CEF in study region.

**Table 1:** Degree of CEF in sampled regions estimation.

	**Shaanxi Province**			**The Ningxia Hui Autonomous Region (Ningxia Province)**		
**Year**	**Financial development (Loan-to-deposit ratio)**	**Environmental sustainability** **(Environmental regulation)**	Coordination degree*100	**Financial development** **(Loan-to-deposit ratio)**	**Environmental sustainability (Environmental regulation)**	Coordination degree*100
2013	0.61	0.00	0.89	0.96	0.00	0.56
2014	0.64	0.00	1.10	1.02	0.00	0.87
2015	0.68	0.00	0.98	1.09	0.00	1.13
2016	0.68	0.00	0.90	1.07	0.00	0.69
2017	0.68	0.00	0.73	1.04	0.00	1.01
2018	0.71	0.00	0.66	1.10	0.00	0.58
2019	0.75	0.00	0.64	1.16	0.00	0.55

The * symbol indicates multiplied by.

### Explanation of the Variables and Descriptive Statistics

I) The dependent parameter was the agriculture investment of the farmers *(Invest)*. The research looked at 2 different forms of agriculture investments: (a) Liquid investment that is straightly tied to livestock or agriculture. (b) Whereas the indirectly tied investment to livestock and agriculture is fixed investment.

II) The financial limitations were the mediating variables. Heterogeneity associated with rate of interest, period, method, and other contractual terms of various loans were all taken into account. The influence of farmers' finance over the investment of agriculture couldn't be correctly quantified if the aggregate of many loans was direct. The influence of finance debt over agriculture investment might be exaggerated. A proxy parameter for financing limitations is the proportion of the aggregate loans of farmers to their aggregate household livelihoods resources for the year. 3 aspects were used for measuring financing limitations; total, large, and small finance scale, i.e., *(Tscale), (Lscale)*, and *(Sscale)*.

III) The indicators of GFPs were the key explanatory factors. For the execution of policy, time dummy parameter is indicated by *Time*. We chose a period of 2017 for the policy to take effect. As a result, the year preceding and following 2017 was set as 0 and 1, respectively. The dummy variable *Treated* grouped by the CEFs' degree in the study region, with 1 indicating a high degree of coordination while 0 indicating no coordination.

IV) Following the former research work regarding the agriculture investment of the farmers and financial activities (42–44), many control variables were used in the current research; (a) While looking into the impact of household features over agriculture investment of the farmers, we chose the social capital (SC) and labor structure (Labor) of the peasant household, revenue from agriculture (A-income), and overall revenue of the peasant household (T-income). (b) Agriculture output features were described through cultivated farmland managed by households (Land) and agriculture productivity fix assets (Asset). The influence of disparities in agriculture asset endowment and input on farmers' agriculture output costs was explored. (c) We used transport ease (Transport) and satisfaction of the farmers from the services provided by bank (BS) as well as control for local variations to study the influence of loan features over the agriculture investment of the farmers (Table 2).

**Table 2:** Depiction of estimated variables.

**Variables**	**Indication**	**Description**	**Mean**	**S.D**.	**Mini:**	**Maxi:**
Dependent variable	Invest	Using the logarithm to calculate the amount of money spent on agricultural production goods	8.85	1.44	5.30	15.20
Mediator variables	T-scale	Aggregate finance scale = annual amount of aggregate loan / annual assets of aggregate household	0.13	0.12	0.00	0.83
	L-scale	Portion of overall debt scale that is higher than the average is referred to as large finance scale	0.24	0.09	0.13	0.47
	S-scale	Portion of overall debt scale that is lower than the average is referred to as small finance scale	0.05	0.04	0.00	0.12
Independent variables	Time	GFPs' effectiveness is expressed by dummy variable	0.43	0.50	0.00	1.00
	Treated	Area's CEF level is expressed by dummy variables	0.47	0.50	0.00	1.00
Control variables	Labor	Labor structure of the rural household = Working-age population/Total household population	0.59	0.22	0.14	1.00
	SC	Member of the family is working as: financial institution, govt. sector, or village official value: 0, 1	0.21	0.41	0.00	1.00
	A-income	Farmers per year income/revenue from breeding and farming, as well as the leasing of acreage and agriculture equipment and machinery, is considered agriculture income: unit Yuan	40,558.13	58,603.64	2,000.00	237000.00
	T-income	Income from property, transfer income, wage income, and production income are considered as total income, unit Yuan	84,560.51	82,860.27	15,000.00	36,0000.00
	Land	A family's cultivated land comprises areas for planting trees and crops, unit acres	16.31	19.04	0.50	72.00
	Asset	Livestock, machinery and supplying material conditions for the process of production are considered as fixed assets of agriculture, unit Yuan	9308.61	20,940.10	0.00	80,000.00
	Transport	The ease with which farmers can get to financing organizations that issue loans. Very inconvenient = 1, inconvenient = 2, general = 3, convenient = 4, very convenient = 5	3.88	0.74	1.00	5.00
	BS	Satisfaction of the farmers from the services provided by bank. Very dissatisfied = 1, dissatisfied = 2, general = 3, satisfied = 4, very satisfied = 5	3.98	0.64	1.00	5.00

High coordination region's farmers made up 47.14 percent of the whole sample/data. The average value of agriculture investment was 8.85, with a standard deviation and median of 1.44 and 8.69 respectively. This suggested significant variation in the agriculture investment of the farmers in study site. The estimated value of standard deviation of mediator variable *(Tscale)* was 0.13, whereas maximum 0.83, and average value was 0.09. When median value of large finance scale (0.24) and small (0.04) finance scale were added together, it showed that various financial scales of the farmers and the degree of their finance limitations differed substantially. In order to remove the impact of extremes, all continuous parameters were handled using the Winsorization approach.

### Specification of the Econometric Model

Using DID approaches from the literature as a guide (45), the GFPs' influence over the agriculture investment of the farmers in regions with higher CEF is examined in current research. This DID approach is useful in determining a policy's influence through analyzing the policy's various impacts on the control and treatment groups (46): DID model is employed in observational studies where the control and treatment groups can't be presumed to be interchangeable. It is based on a relatively rigorous concept of exchangeability, i.e., in other words, in the lack of treatment, the unobserved variations between the control and treatment groups are identical throughout time. Whereas, it requires panel data or repeated cross sectional data. Similarly, the inclusion of multiple time periods and calculation of standard errors are easy in this model, as well as other variables can be controlled in order to prevent unreliable coefficient estimations (47). Mathematically, it is stated as:


(3) $$\begin{array}{l} Invest_{it}=\alpha_{0}+\alpha_{1}Time_{it}+\alpha_{2}Treated_{it}+\alpha_{3}Time_{it}\times Treated_{it}\\ +\alpha_{n}Controls_{it}+\sum Year+\sum Reg+\varepsilon_{it} \end{array}$$

In the above Equation 3, where farmers and years are demonstrated through *i* and *t* respectively. The agriculture investment of the farmers is *Invest* while the dummy variable for GFP's time is expressed by *Time*. Similarly, the areas (sampled areas) having various degrees of CEF is expressed by *Treated*, control variables representation is *Controls* Random error terms of same distribution and independent property are grouped together as ε. Representation of annual dummy variable is *Year*. Dummy variable of the sample area is denoted by *Re*_*g*_, this indicates the year, as well as the impact of the sample regions. The DID model's fundamental independent variable is the *Time*×*Treated*, i.e., interacting term. It reflects GFPs' influence over the agriculture investment of the farmers in regions with higher CEF.

The current research developed a model of mediating effect (Equations 4 and 5) by following (16), in order to examine the financing limitations' mediating process on the influence of GFPs on the agriculture output cost of farmers.


(4) $$\begin{array}{l} Tscale_{it}\;(or\;Lscale{\;}_{it}\;or\;Sscale\;\;e_{it})=\;\phi {\;}_{0}+\phi_{1}Time_{ij}\times Treated_{it}\\ +\phi_{n}Control_{it}+\sum Year+\sum Reg+v_{it} \end{array}$$

(5) $$\begin{array}{l} Invest_{ti}=\gamma_{0}\;+\;\gamma_{1}\;Time_{it}\times Treated_{it}\\ +\gamma_{2}Tscale_{it}(orLscale_{it}\;or\;Sscale\;e_{it})\\ \begin{array}{c} +\gamma_{n}Controls_{it}+\sum^{\text{​}}Year\;+\;\sum^{\text{​}}Reg+\mu_{it} \end{array} \end{array}$$

In which, various financial scales are represented by *(Tscale)* (total), *Lscale* (large), and *Sscale* (small scale finance), that demonstrates various credit capabilities and financial limitations of the farmers. Farmers with high levels of finance (*Lscale*) and good credit capabilities would have fewer financial limitations, whereas those having small levels of finance *(Sscale)* and weak credit capabilities would have stronger financial limitations. The random error terms for both models (presented in Equations 4 and 5) are shown by ν and μ respectively. Moreover, following are the steps for detecting mediation effect; (a) After reviewing the assessed coefficient α_3_ in third model (Equation 3) and coefficient α_3_ is statistically positive and significant then there is a direct effect over the agriculture investment of the farmers in the regions with high CEF from GFP. (b) After examining the assessed coefficients *(*ϕ_1_*)* and *(*γ_1_ *and* γ_2_*)* from both models (presented in Equations 4 and 5) respectively, and when these coefficients are found significant then it reflects partially mediating effect. Similarly, when γ_1_ doesn't show statistically significance while ϕ_1_ and γ_2_ both are found significant then this reflects complete mediation effect. In addition, analysis of bootstrap is applied when any of the coefficients in both models (presented in Equation 4 and 5) is statistically not significant.

## Estimated Outcomes and Discussion

### Estimated Outcomes of the DID Regression

The following Table 3 represents the estimated regression findings obtained through DID model. The Tobit regression model was employed in the current research to minimize data-induced deviations. The computed coefficients of the main independent parameter (*Time*×*Treated*) are significantly positive [α_3(1)_ = 1.25, t=13.02; α_3(2)_ = 0.14, t = 1.90; α_3(3)_ = 0.16; t = 2.45], as shown in column 1 to column 3. The first hypothesis is confirmed through the aforementioned outcomes, i.e., there is stimulating impact of GFPs over the agricultural investment of farmers in regions with high CEF. Farmers might be encouraged to minimize their use of both high polluting and high energy utilizing agricultural techniques as a result of the introduction of GFPs. The farmers living in higher CEF regions may efficiently promote production's low carbon and green development trend. This permits for better resource consumption and productivity improvement in viable advancement.

**Table 3:** Estimated outcomes of the DID regression.

	**(1)**	**(2)**	**(3)**
**Variables**	**Invest**	**Invest**	**Invest**
Time	0.82***	0.04	0.06
	(12.55)	(−0.51)	(−0.56)
Treated	0.05	0.04	0.13
	(0.81)	(−0.97)	(−0.43)
Time*Treated	1.25***	0.14*	0.16*
	(13.02)	(1.90)	(2.45)
Labor		−0.45***	−0.32***
		(−5.71)	(−5.73)
SC		0.18***	0.28***
		(4.71)	(3.71)
A–income		7.7E−06***	6.7E−06***
		(14.35)	(14.40)
T–income		1.9e−06***	3.8e−06***
		(5.67)	(5.68)
Land		0.00***	0.01***
		(6.48)	(6.50)
Asset		5.44e−06***	3.1e−06***
		(6.57)	(4.32)
Transport		−0.06***	−0.04***
		(−2.75)	(−1.58)
BS		−0.06**	−0.04**
		(−2.24)	(−1.48)
year		0.04**	0.05**
		(2.53)	(2.54)
Reg		0.60***	0.63***
		(12.23)	(12.26)
Constant	8.70***	−69.75**	−75.70**
	(196.03)	(−2.26)	(−3.24)
			
Observations	2,350	2,350	2,350
R–squared	0.11	0.63	0.30

S.E in (), ***, **, significant at 1, 5, and 10% respectively.

### Estimated Outcomes of Mediation Test

This research evaluates models (3, 4) to investigate the influence of financing limitations on linkage b/w agriculture investment by farmers and GFP (Table 4). The full sample estimate findings are in column-1 through column-3 and the comparison evaluation outcomes are in column-4 through column-7, classified by finance scale. According to the assessed outcomes that GFPs have statistically significant positive impact on the agriculture investment of farmers. There is significantly adverse influence of GFP on finance scales of farmers [ϕ_1(*Tscale*)_ = −0.02, t = −2.26; ϕ_1(*Lscale*)_ = −0.03, t = −2.72] in group with large finance scale and full sample. Which suggests the adverse influence of GFP over financial scales of farmers. The financial scales of the farmers and their financing limitations are decreased and increased respectively due to the deployment of green finance. After incorporating intermediate parameter (*Tscale, Lscale*, and *Sscale*), the computed coefficient in financial scale in 3rd column is adverse [γ_2(*Tscale*)_ = −0.45, t = −2.90] while those of *Time*×*Treated* declines [α_3_ = 0.14, t = 1.90; γ_1(*Tscale*)_ = 0.1315, t = 1.77]. It suggests the mediation influence of financial scale between agriculture investment and GFP in a partial opposite way. In comparison to the immediate and positive influence of GFPs over agriculture investment, the addition of a financial scale parameter reduces the favorable influence of GFPs over agriculture investment. The investment of farmers regarding agriculture production is reduced due to financial limitations. The aforementioned findings apply to locations with a high CEF. In 6th column, where regression coefficient is not statistically significant, i.e., [ϕ_1(*Sscale*)_ = −0.00, t = −0.50] in the empirical outcomes of financial scales' grouping. The small finance scale's indirect effect coefficient as per the outcomes of 1000 samples through bootstrap analysis was statistically not significant −0.00 (*p* = 0.650). Which suggests the instability of small finance scale's intermediary impact b/w agricultural investment and GFPs. The second and third hypotheses are supported by the assessed outcomes of Table 4. While encouraging the farmers to improve their agriculture productivity, the capital restrictions can effectively limit the deployment of GFP. With the increasing financing restrictions, farmers' ability to allocate green financing credit in agriculture production becomes more difficult. This is especially apparent in locations where environmental and financing advancement are more thoroughly integrated.

**Table 4:** Estimated outcomes of mediation test.

	**Benchmark**	**Total–finance scale**		**Large–finance scale**		**Small–finance scale**	
	**(1)**	**(2)**	**(3)**	**(4)**	**(5)**	**(6)**	**(7)**
**Variables**	**Invest (Path c)**	**T–Scale** **(Path a)**	**Invest (Paths b and c')**	**L–scale**	**Invest**	**S–scale**	**Invest**
Time*Treated	0.14*	−0.02**	0.13*	−0.03***	0.31***	−0.00	0.13*
	(1.90)	(−2.26)	(1.77)	(−2.72)	(3.10)	(−0.50)	(1.82)
T–scale			−0.46***				
			(−2.9)				
L–scale					−0.90***		
					(−3.23)		
S–scale							1.34***
							(2.75)
Labor	−0.45***	−0.00	−0.45***	0.018	−0.54***	−0.00	−0.41***
	(−5.71)	(−0.90)	(−5.77)	(1.19)	(−4.20)	(−0.40)	(−4.26)
SC	0.18***	0.02***	0.191***	0.03***	0.28***	−0.01***	0.15***
	(4.71)	(3.34)	(4.90)	(3.96)	(4.70)	(−3.94)	(2.79)
A–income	7.7e−06***	−1.2e−08	7.8e−06***	2.01e−07*	3.37e−06***	−5.81e−08	1.11e−05***
	(14.35)	(−0.17)	(14.36)	(1.90)	(3.63)	(−1.65)	(16.68)
T–income	1.9e−06***	−1.2e−07***	1.8e−06***	−1.55e−08	2.81e−06***	−1.10e−08	1.83e−06***
	(5.67)	(−2.90)	(5.50)	(−0.23)	(4.71)	(−0.54)	(4.75)
Land	0.01***	0.00***	0.01***	−0.00	0.00***	0.00*	0.01***
	(6.48)	(3.70)	(6.69)	(−0.91)	(2.60)	(1.78)	(5.84)
Asset	5.5e−06***	1.52e−08	5.5e−06***	−3.2e−08	5.3e−06***	−6.9e−08	6.5e−06***
	(6.58)	(0.20)	(6.60)	(−0.21)	(3.90)	(−1.32)	(6.43)
Transport	−0.06***	0.01***	−0.06**	−0.00	−0.22***	0.01	0.03
	(−2.76)	(2.81)	(−2.60)	(−0.90)	(−5.90)	(0.70)	(1.12)
BS	−0.06**	0.01**	−0.05**	0.00	0.11**	−0.00***	−0.13***
	(−2.30)	(2.60)	(−2.10)	(1.08)	(2.50)	(−2.70)	(−4.12)
Year	0.04**	0.01***	0.04***	0.00***	0.04***	−0.00	0.05***
	(2.60)	(4.80)	(2.81)	(3.60)	(2.80)	(−1.22)	(4.30)
Reg	0.60***	0.016**	0.61***	−0.05***	0.83***	0.02***	0.41***
	(12.30)	(2.50)	(12.40)	(−5.40)	(9.50)	(7.80)	(7.25)
Constant	−69.75**	−18.60***	−78.21**	−11.10***	−73.72**	1.56	−93.01***
	(−2.30)	(−4.80)	(−2.53)	(−3.50)	(−2.50)	(1.30)	(−3.94)
R–squared	0.63	0.09	0.63	0.073	0.61	0.11	0.68

S.E in (), ***, **, * significant at 1, 5, and 10% respectively.

The term “green finance” refers to a scenario where environmental protection and finance are merged. Subsidies from the government are among the highly successful approaches for encouraging green development and lowering the emission of carbon dioxide and improving environmental quality (48). It is intended, on one side, to finance the development of innovative products, such as, Huang et al. (49) applied a game model between firm, bank, and government, which revealed that government subsidies are a beneficial interference for improving quality of the environment. While, on other side, it's employed to comply with environmental regulations and cut carbon emissions (50, 51).

### Management Type Heterogeneity's Impact Based on Mediation Test Analysis

The portion with a negligible mediation impact is eliminated in current investigation (Small financial scale group). Three subgroups were made of farmers based on different agriculture management' compositions in their residential management: Purely agricultural, agricultural/farm oriented & simultaneously functioning others, and non-farm/agricultural oriented & simultaneously functioning agriculture (also change them in Table 5). From the perspective of farmers' heterogeneity management methods, Table 5 indicates the variations in financing restrictions' mediating impacts. The farmers with non-farm/agricultural oriented operating style, according to the estimated outcomes, shown much larger assessed coefficient as compared to other management groups. The restriction of finance has an entirely mediated influence. The estimated outcomes from 1000 samples through bootstrap analysis revealed that coefficients of indirect impact for other management groups were −0.02 (*p* = 0.65) and −0.001111 (*p* = 0.98), respectively. Which signified that intermediate impact of other groups of farmers' financing restrictions in the association b/w agriculture investment and GFP isn't acknowledged.

**Table 5:** Management type heterogeneity's impact based on mediation test analysis.

	**Purely agriculture**		**Agricultural/farm oriented**		**Non–farm/agricultural oriented**	
	**(1)**	**(2)**	**(3)**	**(4)**	**(5)**	**(6)**
**Variables**	**T–Scale (Path a)**	**Invest (Paths b and c')**	**T–scale**	**Invest**	**T–scale**	**Invest**
Time*Treated	−0.056***	0.91***	0.00	0.03	−0.02***	0.11
	(−3.60)	−7.95	−0.14	−0.08	(−4.32)	−1.33
T–scale		−0.37		−0.28		−1.40***
		(−0.88)		(−0.44)		(−4.63)
Labor	0.02	−0.96***	−0.06	0.15	0.117**	−0.74***
	−0.79	(−4.83)	(−1.52)	−0.45	−1.05	(−4.52)
SC	0.041***	0.18**	−0.02	0.38**	0.05***	0.41***
	−4.34	(−2.53)	(−1.03)	−2.10	−3.99	−5.57
A–income	0.00	1.2e−05***	1.2e−06***	1.1e−05**	0.00	2.6e−05***
	−0.59	−4.79	−2.84	−2.80	−0.56	−10.88
T–income	0.00	0.00	−4.9e−07**	−5.5e−06**	0.00	0.00
	(−0.52)	(−1.04)	(−2.32)	(−2.92)	(−1.04)	−1.46
Land	0.00	0.02***	−0.01***	0.023***	−0.01**	−0.01
	−1.24	−10.14	(−3.40)	−3.82	(−2.86)	(−1.44)
Asset	0.00	−1.5e−06***	0.00	−9.4e−06*	0.00	2.3e−05***
	−1.21	(−9.54)	−0.10	(−1.77)	(−0.91)	−3.66
Transport	−0.01	0.26***	−0.02*	−0.49***	0.01	−0.04
	(−1.16)	−4.48	(−1.96)	(−6.67)	−1.39	(−0.90)
BS	0.04***	0.28***	0.01	0.36***	−0.03***	0.16***
	−4.37	−4.15	−1.27	−3.96	(−2.78)	−2.83
Year	0.01***	0.00	0.01	0.02	0.01*	0.03
	−4.59	−0.31	−1.29	−0.38	−1.88	−1.45
Reg	0.01***	0.52***	−0.04	1.06***	−0.03*	0.46***
	(−3.95)	−3.09	(−5.39)	−4.50	(−2.10)	−4.50
Const:	−19.11***	−3.55	−3.68	−38.67**	−11.23*	−48.66***
	(−4.56)	(−0.12)	(−0.27)	(−0.30)	(−1.83)	(−2.90)
R–squared	0.23	0.87	0.19	0.47	0.16	0.47

S.E in (), ***, **, * significant at 1, 5, and 10% respectively.

Its economic consequence is that financing restrictions of non-agricultural farmers have a stronger adverse regulatory impact b/w GFP and agriculture investment as compared to other farmer management groups in high CEF regions. This represents the fact that non-agriculture farmers are the intended participants and beneficiaries of the existing GFP. Whereas 4th hypothesis is supported by the estimated outcomes.

### Analysis of Robustness Test

#### Analysis of the Parallel Trend Test

In order to analyze the parallel tendency, the current research utilizes the technique of event-study, making sure that all investigation items exhibit identical tendency prior to policies execution (52). The current research establishes the dummy parameters of yearly impact before and after 2017 using 2017 as benchmark. Then, for comparing plan execution before and after, create the interaction object of year virtual variable and empirical grouping *(Treated)* virtual variable. The policy differs significantly depending on the study object as shown in Table 6. Prior to the establishment of green finance policy, the 2 groups' tendency is essentially identical. This demonstrates that the preceding regression outcome and sample categorization are correct.

**Table 6:** Estimated outcomes of parallel trend test.

**Variables**	**Coef**.	**St. Err**.	***T*-value**
Before_4	0.06	−0.08	0.72
Before_3	0.05	−0.06	0.73
Before_2	0.02	−0.08	0.27
Before_1	0.02	−0.06	0.30
After_1	0.22***	−0.08	2.65
After_2	0.19**	−0.09	2.16
Labor	−0.45***	−0.08	−5.71
SC	0.18***	−0.04	4.71
A–income	7.7e−06***	0.00	14.34
T–income	1.8e−06***	0.00	5.66
Land	0.01***	0.00	6.48
Asset	5.4e−06***	0.00	6.56
Transport	−0.06***	−0.02	−2.75
BS	−0.06**	−0.03	−2.24
Year	0.05***	−0.01	4.24
Reg	0.61***	−0.06	10.31
Constant	−84.33***	−21.85	−3.86
Obs:	2350.00		
R–squared	0.63		

S.E in (), ***, ** significant at 1, 5, and 10% respectively.

#### Analysis of the PSM-DID Assessment

The current research utilizes propensity score matching technique with DID models (PSM-DID) by following (16, 47, 53) in order to examine the variation between control and empirical groups. The agriculture investment of farmers and control variables' propensity score as covariates were determined. The estimated outcomes of matching test in Table 7 where mostly variables have *P*-value < 0.1, bias is <10%, and statistically insignificant *T*-test, suggesting that after matching, there is little difference between the two groups. Therefore, PSM can be used successfully. The estimated outcomes in Table 8 for PSM-DID shows significantly active coefficient for *Time*×*Treated* (*a*_3_= 0.31, t = 2.66). The rest of variables, with the exception of BS variable, have all passed assessment. The rationale for selecting the control variable can be observed. This suggests the preceding regression outcome and control variables' selection are appropriate.

**Table 7:** Estimates of PSM–DID effectiveness test.

**Variables**	***T*–test**	***P*–value**	**Bias (%)**
Labor	−1.14	0.00	−4.6
SC	−1.91	0.96	−7.9
A–income	−0.54	0.00	−1.9
T–income	−0.96	0.70	−3.6
Land	−0.56	0.00	−2.0
Asset	−0.13	0.88	−0.5
Transport	1.01	0.00	4.4
BS	−0.94	0.00	−4.0

**Table 8:** Assessments of PSM–DID.

**Variables**	**Coefficient**	**S.E**	**T–ratio**	**p**
Time	−0.16	0.09	−1.67	0.09
Treated	−0.01	0.05	−0.16	0.87
Time*Treated	0.31	0.11	2.66	0.01
Labor	−0.43	0.10	−4.15	0.00
SC	0.19	0.05	3.91	0.00
A–income	0.00	0.00	9.65	0.00
T–income	0.00	0.00	4.93	0.00
Land	0.01	0.00	5.86	0.00
Asset	0.00	0.00	5.36	0.00
Transport	−0.09	0.03	−3.36	0.00
BS	−0.05	0.03	−1.36	0.17
Year	0.06	0.02	2.96	0.00
Reg	0.64	0.06	10.88	0.00
Constant	−109.72	39.91	−2.75	0.01
R–squared	0.66			

The * symbol indicates multiplied by.

#### Alternative Variables

At last, in order to execute robustness test, the goal of current research is to change the fundamental variable. Increasing the financial extent of farmers is a critical step for improving agriculture output finances and boosting income of farmers. The importance of removing financing obstacles to revenue growth of farmers, and the interpretable variables have the value [0, 1]. The current research, by following (16, 54), investigated the intermediate impact through standardizing the new mediator variables applying the proportion of aggregate loans and yearly income/revenue as an alternate measure of financial scale. Total scale, large scale, and small scale finance, i.e., *(tscale), (lscale)*, and (sscale) respectively, are used to examine new mediator variable.

Table 9 where regression outcomes reveal significant positive impacts of GFPs over the growing costs of agriculture output [γ_1(tscale)_ = 0.14, t = 1.83; γ_1(lscale)_ = 0.22, t = 2.21]. GPFs have shown adverse impacts (in 2nd column and 4th column) over removing finance constraints for farmers in the regions of high CEF [ϕ_1(*tscale*)_ = −0.04, t = −1.87; ϕ_1(*lscale*)_ = −0.11, t = −5.65], and the limitations of finance serve as a partial intermediate role. After the inclusion of intermediate variable, the computed coefficient of *Time*×*Treated* also declines, suggesting that mediator variables appear to be preventing GFPs from having a favorable influence on the agriculture investment of farmers. The estimated indirect impact's coefficient was −0.00 (*p* = 0.54) when coupled with bootstrap analysis, suggesting that intermediate impact of small finance scale is inadequate. The findings support the findings of the study's previous estimation.

**Table 9:** Estimated outcomes of robustness test (alternative variable).

	**Benchmark**	**Total–finance scale**		**Large–finance scale**		**Small–finance scale**	
	**(1)**	**(2)**	**(3)**	**(4)**	**(5)**	**(6)**	**(7)**
**Variables**	**Invest (Path c)**	**t–scale (Path a)**	**Invest (Paths b and c')**	**l–scale**	**Invest**	**s–scale**	**Invest**
Time*Treated	0.14*	−0.04*	0.14*	−0.11***	0.22**	−0.01	0.13*
	−1.90	(−1.87)	−1.83	(−5.65)	−2.21	(−0.66)	−1.85
t–scale			−0.1243*				
			(−1.7)				
l–scale					−1.09***		
					(−6.69)		
s–scale							0.37***
							−3.45
Labor	−0.49***	−0.09***	−0.46***	−0.054**	−0.62***	−0.08***	−0.38***
	(−5.71)	(−3.76)	(−5.83)	(−2.16)	(−4.84)	(−3.33)	(−3.99)
SC	0.18***	0.02	0.19***	−0.01	0.25***	−0.01	0.14***
	−4.71	−1.38	−4.76	(−0.69)	−4.26	(−1.00)	−2.62
A–income	7.7e−06***	−3.7e−07**	7.7e−06***	0.00	3.1e−06***	−6.1e−07***	1.1e−05***
	−14.35	(−2.41)	−14.26	(−0.29)	−3.44	(−3.75)	−16.86
T–income	1.9e−06***	−1.0e−06***	1.8e−06***	−9.7e−07***	1.8e−06***	−7.8e−07***	2.1e−06***
	−5.67	(−10.70)	−5.17	(−8.47)	−2.91	(−8.28)	−5.34
Land	0.01***	0.01***	0.01***	−0.01**	0.00**	0.00***	0.01***
	−6.48	−4.84	−6.63	(−2.43)	−2.21	−6.08	−5.38
Asset	5.4e−06***	9.5e−07***	5.6e−06***	2.1e−06***	7.5e−06***	7.1e−07***	6.1e−06***
	−6.57	−4.03	−6.69	−8.05	−5.48	−2.92	−6.06
Transport	−0.06***	0.02***	−0.06***	0.01**	−0.20***	0.00	0.03
	(−2.75)	−2.86	(−2.64)	−2.06	(−5.44)	0.00	−1.16
BS	−0.06**	0.00	−0.06**	−0.02**	0.08*	−0.01	−0.13***
	(−2.30)	−0.60	(−2.33)	(−2.40)	−1.89	(−1.50)	(−4.20)
year	0.04**	0.02***	0.05***	0.01**	0.04***	−0.00*	0.05***
	−2.60	−3.69	−2.70	−2.89	−3.04	(−1.70)	−4.40
Reg	0.60***	0.04**	0.61***	−0.14***	0.72***	0.07***	0.43***
	−12.30	−2.30	−12.29	(−8.69)	−8.20	−4.68	−7.50
Cons:	−69.76**	−32.17***	−73.75**	−15.97***	−80.32***	9.99*	−94.54***
	(−2.30)	(−3.70)	(−2.40)	(−2.80)	(−2.69)	−1.69	(−4.01)
R^2^	0.63	0.18	0.63	0.31	0.61	0.23	0.68

S.E in (), ***, **, * significant at 1, 5, and 10% respectively.

Furthermore, the estimated regression outcomes of Table 10 where mediation test's mediator variables of farmer management types' heterogeneity were also altered. The assessed outcomes endorsed that non-agriculture management has significantly influenced the mediating impact of finance limitations. With the exception of non-farm/agricultural oriented operation type's finance limitations, which had a fully mediating impact, the rest of the groups are failed in this mediating impact. Based on bootstrap analysis, the estimated coefficients of indirect effect for other management types were −0.07 (*p* = 0.14) and −0.05 (*p* = 0.49) respectively. The finance limitations of farmers have an intermediate impact on other types of management isn't acknowledged. This indicates that the study's findings are, to a degree, trustworthy.

**Table 10:** Estimated outcomes of robustness test (management type heterogeneity).

	**Purely agriculture**		**Agricultural/farm oriented**		**Non–farm/agricultural oriented**	
	**(i)**	**(ii)**	**(iii)**	**(iv)**	**(v)**	**(vi)**
**Variables**	**t–scale (Path a)**	**Invest (Paths b and c')**	**t–scale**	**Invest**	**t–scale**	**Invest**
Time*Treated	−0.20***	0.67***	−0.13*	−0.04	−0.08**	0.24
	(−5.13)	−5.16	(−2.68)	(−0.14)	(−2.70)	−1.50
t–scale		−1.63		−0.38		−0.98***
		(−0.62)		(−0.91)		(−3.94)
Labor	−0.06	−1.04***	−0.35***	0.03	0.02	−0.65***
	(−1.07)	(−5.91)	(−5.96)	−0.09	−0.78	(−4.00)
SC	−0.0378**	−0.25***	0.04	0.40**	0.01	0.40***
	(−2.03)	(−4.05)	−1.15	−2.18	−0.60	−5.60
A–income	3.1e−06***	1.7e−05***	0.00	1.1e−05**	0.00	2.5e−05***
	−4.34	−7.07	(−1.30)	−2.66	(−1.07)	−9.53
T–income	−3.9e−06***	−8.8e−06***	0.00	−5.3e−06**	−2.1e−06***	0.00
	(−5.60)	(−3.60)	(−0.01)	(−2.88)	(−11.81)	(−1.00)
Land	−0.00***	0.01***	0.00	0.02***	0.00***	−0.01*
	(−2.62)	−9.83	(−0.40)	−4.10	−4.51	(−1.91)
Asset	3.8e−07***	−9.6e−07***	2.2e−06**	0.00	−5.2e−06***	2.3e−05***
	−8.47	(−5.85)	−2.45	(−1.65)	(−4.34)	(−3.56)
Transport	−0.04**	0.21***	0.01	−0.48***	0.00	−0.10*
	(−2.29)	−3.94	−080	(−6.59)	(−0.04)	(−1.96)
BS	−0.02	0.23***	−0.02	0.34***	0.00	0.15**
	(−1.14)	−3.91	(−1.4)	−3.90	(−0.10)	(−2.69)
year	0.016**	0.05*	0.01*	0.08**	0.01	0.03
	−2.20	−1.90	−1.90	−2.20	(−0.89)	(−0.79)
Reg	−0.29***	0.08	−0.26***	0.99***	−0.09***	0.43***
	(−7.80)	−0.70	(−6.29)	−3.90	(−4.20)	(−4.01)
Constant	−30.73**	−83.27*	−21.23	−144.39**	−10.50*	−46.30*
	(−2.10)	(−1.69)	(−1.80)	(−2.10)	(−1.90)	(−1.69)
R–squared	0.58	0.90	0.57	0.46	0.45	0.48

S.E in (), ***, **, * significant at 1%, 5% and 10 % respectively.

## Concluding Remarks and Policy Recommendations

### Conclusions

This study draws the following conclusions:

(1) GFPs' implementation promotes farmers' financing methods' diversification and provides some effective support for farmers' investment in agriculture productivity. It inspires peasants to capitalize in green, low-carbon productivity techniques and to improve their skill.
(2) The implementation of GFPs limits peasants' financial support somewhat, and the increase in financial restrictions has a negative impact on farmers' agricultural input. This further restricts the promotion of farmers' agricultural production optimization. The practices seem to be more likely to affect the farming financial behavior of peasants with greater investment levels in the intermediating directions of the impact of GFP on peasants' investment through budgetary restrictions. GFPs' implementation makes the green production of farmers' subject to stronger behavioral incentives, peasants are being forced to continually increase the operational expenses of agriculture productivity upgrading. Whether farmers conduct green production becomes a necessary condition for them to obtain financial support more easily in the ecofriendly monetary sector. GFPs will increase the admittance threshold of farmers' credit. Agricultural green production projects with significant environmental impacts increase the hollowing impact of ecological regulatory expenses on peasants' investment on production, putting peasants in a bind.
(3) Peasants whom was primarily engaged in non-agricultural operations may be more likely to benefit from GFPs. People seem to be more vulnerable to adverse adaptation impact of income restrictions on the policy-agriculture investment relationship. Non-agricultural peasants do have pressing requirements for green-finance and bear a greater portion of expenses of environmental compliance. It magnifies the promotion effect of green-finance development on non-agricultural planning. GFPs' financial assistances for agriculture productivity activities are insufficient. The rising expense of compliance with ecological standards decrease peasants' optimistic reaction to GFPs' production operations. GFPs' real effects deviate from the policy objectives.

The currently implemented GFPs have limited guidance in rural areas, especially in the transformation and upgrading of farmers' sustainable agricultural production. The benefit of “rein” was not highlighted, and it has been diminished to overall lending financial institutions. The efficient supply provided by GFPs lags behind the demand for green agricultural development in rural areas and reduces farmers support for green agricultural development. These findings are most evident in areas with a high coordination between environmental sustainability and financial development. Despite the fact that this investigation has acknowledged a comprehensive interpretation of identifying the influence of green financial policies and finance limitations on the agriculture investment of the farmers in terms of farmers' heterogeneity to achieve sustainable development in Ningxia and Shaanxi provinces. Nevertheless, the findings of the study demonstrated the significance of the aforementioned elements. This research is quantified based on the Ph.D. dissertation's single goal, and we are still working on exploring the current topic in many aspects. However, as mentioned earlier that these are the fast-growing areas of western China in many aspects and are the major grain producing areas. Similarly, efficient establishment of green finance in the areas of western China is a critical linkage in achieving the green economic revolution. Therefore, from future perspective, further investigation is needed regarding the significance of current topic through advanced econometric approaches, and their implementation in future policy guidelines.

### Policy Recommendations

(1) Government and regulatory agencies should improve guidance and incentive systems. To promote the development of diverse green financial markets, a small operating system of GFPs supporting agricultural green development ought to be built. Green financial services' overall assessment system and target requirements should be improved. Financial support to banks that provide green financial services should be provided. It will reduce their operative threats and costs and increase their enthusiasm for green finance services. To advocate for the importance of green and carbon neutral agricultural production for farmers, environmental protection should be promoted. Farmers should fulfill their social responsibilities and improve their responsiveness to policy. (2) Financial institutions should accelerate the development of green financial products to gradually meet the multi level procedure of farming circular markets and the diverse funding needs of farmers. Financial institutions should: intensify efforts to radiate green finance services to rural areas; reduce financing cost of green projects; improve the coverage, availability and convenience of green financial services; effectively solve the credit constraints in green agricultural production, and increase the “green” benefits of policies.

## Data Availability Statement

The raw data supporting the conclusions of this article will be made available by the authors, without undue reservation.

## Ethics Statement

The studies involving human participants were reviewed and approved by Northwest A&F University. Written informed consent for participation was not required for this study in accordance with the national legislation and the institutional requirements.

## Author Contributions

BM: conceptualization, formal analysis, investigation, software, methodology, data curation, writing—original draft, and writing—review and editing. AK: investigation, software, methodology, and writing—review and editing. SK: methodology and writing—review and editing. MA: data curation and writing—review and editing. JL: funding acquisition, project administration, and supervision. All authors contributed to the article and approved the submitted version.

## Funding

This paper is supported by Research on the Effectiveness Evaluation, Risk Control and System Construction of the Agricultural Credit Guarantee Policy, National Natural Science Foundation of China (NSFC), Jan 2019-Dec 2022, No. 71873100. Sponsor and Host: JL. This paper is also supported by Research on the Policy Orientation and Implementation Path of Financial Empowerment of Rural Revitalization, the Soft Science Project of the Central Agricultural Office and the Rural Revitalization Expert Advisory Committee of the Ministry of Agriculture and Rural Affairs, 2022.5.31-2023.5.31, No. rkx20221801, Sponsor and host: JL. This paper is also supported by Rural Revitalization Financial Policy Innovation Team, Chinese Universities Scientific Fund, 2022.1-2023.12, No. 2452022074, Sponsor and Host: JL.

## Conflict of Interest

The authors declare that the research was conducted in the absence of any commercial or financial relationships that could be construed as a potential conflict of interest.

## Publisher's Note

All claims expressed in this article are solely those of the authors and do not necessarily represent those of their affiliated organizations, or those of the publisher, the editors and the reviewers. Any product that may be evaluated in this article, or claim that may be made by its manufacturer, is not guaranteed or endorsed by the publisher.
